# Editorial: Bone health and disease in veterinary species

**DOI:** 10.3389/fvets.2023.1290149

**Published:** 2023-09-20

**Authors:** Alvaro Wehrle-Martinez, Keren Dittmer, Chris W. Rogers

**Affiliations:** ^1^School of Veterinary Sciences, Massey University, Palmerston North, New Zealand; ^2^School of Agriculture and Environmental Sciences, Massey University, Palmerston North, New Zealand

**Keywords:** bone fracture, bone repair, bone histology, bone radiography, computed tomography, Raman, FTIR spectroscopy

The days when bone was considered a static tissue providing only mechanical support for the body are long gone. In fact, bone is a highly dynamic tissue with numerous interactions and functions that provide not only movement, support, and protection but also influence in mineral metabolism and homeostasis, hematopoiesis, immune activity, glucose/energy metabolism, and the endocrine system (1, 2).

The articles within this Research Topic addressed the changes and response of bone to trauma and repair using a variety of tools and techniques. For example, Bow et al. explored and characterized bone repair after trauma using radiography, computed tomography, histology, and biomechanical data. The authors also provided valuable information on the metabolic profile of bone repair using ultra-high-performance liquid chromatography-high resolution mass spectrometry which revealed distinct patterns of small molecule profiles associated with cell differentiation/function and changes in the extracellular matrix conformation that occur during bone healing.

Leal et al. compared five radiographic scoring systems to assess bone healing following tibial plateau leveling osteotomy. They identified that the Visual analog scale, which was 0–100-point modification of the SUB5 scale was the easiest to use and had the greatest repeatability between assessments. An interesting and practical application of the work was the identification of the impact limb positioning had on the assessment of healing irrespective of the scoring system used.

Campeiro Junior et al. using radiography and histology, investigated the effects of cefazolin loaded bone cement in the induced membrane formation technique for healing a segmental bone defect using a chicken radius. The imaging and histological examination of the induced membrane demonstrated cefazolin did not affect thickness of the induce membrane but did moderate some of the histological measurements.

Finally, Wehrle-Martinez et al. presented data collected using Raman and Fourier transform infrared spectroscopy on bone samples from dairy cows affected by catastrophic spontaneous humeral fractures in New Zealand. The analysis provided insight into the increased bone remodeling observed in the humerus of heifers affected with catastrophic spontaneous humeral fractures. This information provided valuable insight into the pathogenesis of this condition and potential temporal sensitivity of the osteoporosis associated with this condition.

These papers published in this Research Topic show that bone, like any other tissue in the body, can be assessed and studied using numerous tools and techniques, and the data obtained provides valuable information into bone structure and function and how varied species respond to bone trauma and repair.

## Author contributions

AW-M: Writing—original draft, Writing—review and editing. KD: Writing—review and editing. CR: Writing—review and editing.
